# Factor structure, measurement invariance, and concurrent validity of the Patient Health Questionnaire-9 and the Generalized Anxiety Disorder scale-7 in a Norwegian psychiatric outpatient sample

**DOI:** 10.1186/s12888-022-04101-z

**Published:** 2022-07-11

**Authors:** Martin Brattmyr, Martin Schevik Lindberg, Stian Solem, Odin Hjemdal, Audun Havnen

**Affiliations:** Department of Psychology, Norwegian University of Science and Technology, Trondheim, Norway

**Keywords:** PHQ-9, GAD-7, Factor structure, Measurement invariance, Reliability, Validity

## Abstract

**Objective:**

The aim of this study was to test factor structure, measurement invariance, and concurrent validity of the nine item Patient Health Questionnaire-9 (PHQ-9) and the seven item Generalized Anxiety Disorder scale-7 (GAD-7) in a heterogeneous outpatient sample.

**Method:**

Outpatients completed the PHQ-9, GAD-7, and the Working Social Adjustment Scale (WSAS) before starting treatment. Study design was cross-sectional, with convenience sampling. The total sample consisted of 831 participants (61% women).

**Results:**

Both PHQ-9 and GAD-7 demonstrated better fit statistics with two-factor and bifactor solutions consisting of a cognitive and somatic factor. Omega hierarchical was .78 for PHQ-9 and .81 for GAD-7. Both instruments achieved scalar invariance across gender, diagnosis, and comorbidity. However, the somatic factors demonstrated poor discriminant validity. These factors are not well separatable and risks being too similar if used together. The general factors of both instruments were most associated with functional impairment, although PHQ-9 demonstrated a stronger association with WSAS (*γ* = .74, *r*^2^ = .62) than GAD-7 (*γ* = .54, *r*^2^ = .32). Using latent mean difference, women and patients with comorbidity had significantly higher scores of both depression and anxiety.

**Conclusion:**

This study shows that the PHQ-9 and GAD-7 may be used as one-dimensional instruments in clinical settings. Tests for measurement invariance supported that both measures are understood and interpreted comparably across gender and diagnostic subgroups.

Standardized outcome measures have been promoted for at least half a century in the mental health field [1]. Two instruments currently at the center of attention are the Patient Health Questionnaire-9 (PHQ-9) [2] measuring depression, and the Generalized Anxiety Disorder scale-7 (GAD-7) [3] measuring anxiety. These instruments have been proposed to be included in core-sets of measures in clinical research [4, 5]. However, these recommendations has also been criticized, amongst other reasons due to conflicting results regarding factor structures, uncertainties about how well the results generalize across groups, and little available knowledge on their transferability to clinical contexts [6]. As a result, there is limited evidence on the adequacy of using these instrument with clinical heterogenous populations, where they also are used the most [6].

Others acknowledge that these instruments are becoming frequently more applied in research and clinical contexts, but emphasizes the importance of measuring other aspects of mental health as well, such as level of functioning [1]. Therefore, factor structures, generalizability across different patient groups, and relationship with functional impairment for PHQ-9 and GAD-7 in adult outpatients with mixed psychiatric disorders will be in focus for this study.

Many different factor structures have been suggested for PHQ-9 [7]. However, the inconsistencies in research findings can be a product of sample properties [8] and methodology [9]. Results from confirmatory factor analysis (CFA) using psychiatric outpatient populations with mixed disorders are sparse. For example, only one out of 33 articles in a recent systematic review included such heterogenous psychiatric outpatient sample [7]. In that particular study, the proposed factor-solution was a two-factor model of the PHQ-9, comprising a cognitive factor and a somatic factor [10]. Still, the usefulness of such two-factor solution has been disputed, amongst others due to a strong correlation between the factors [11]. Therefore, PHQ-9 have been suggested suitable with a bifactor-(S – 1) model assessing patients at risk, or with diabetes in India [12]. This modification of the classic symmetric bifactor model has been proposed as a solution for anomalous results due to single-level sampling and it also increases the interpretability due to using a reference domain [13].

Discussions have been similar regarding GAD-7. For heterogenous outpatient samples, both unitary models constrained with correlated residuals [14, 15], and two-factor solutions have been suggested [16]. The latter study demonstrated a two-factor model of GAD-7 using exploratory factor analysis (EFA), which consisted of a cognitive and a somatic factor, just like previous research on PHQ-9 [16]. Further, GAD-7 has also been suggested suitable with a bifactor-(S – 1) model but limited to the population mentioned above [12].

To justify comparisons between patient groups, tests of measurement invariance (MI) should demonstrate equality of indicator thresholds, or so-called scalar invariance [17]. MI implies restrictions in a hierarchical manner of a model, to point out whether and where properties of an instrument differ across groups. For example, if crying is more strongly associated with depression for women than men, an instrument measuring a latent construct of depression with an item about crying could risk biased results, and assumably not achieve scalar invariance [18]. A systematic review of MI of PHQ-9 presented support for scalar invariance across gender in several studies [7], including a psychiatric outpatient population with mixed disorders [10]. This has also been proposed for GAD-7, in a study with an heterogenous outpatient population [15]. Thus, with heterogenous psychiatric outpatients, both instruments have demonstrated scalar invariance for gender, or so-called gender invariance. However, there is still limited evidence for the Norwegian versions.

In addition to MI, it is important to evaluate the association between symptoms of depression and anxiety with functional impairment, as a way to test their usefulness in clinical contexts. A close relationship between symptoms of depression and anxiety with functional impairment is often implicitly assumed, but rarely tested [19, 20]. However, one review reported a moderate correlation between symptoms of depression and functional impairment [19] and another review reported a weak association between symptoms of anxiety and functional impairment [20]. Accordingly, symptoms of depression seem to be more associated with functional impairment than symptoms of anxiety. One commonly used instrument that measures functional impairment is the Work and Social Adjustment Scale (WSAS) [21]. It has been demonstrated with a unitary factor structure and scalar invariance across gender [22]. Studies have reported higher correlation between WSAS and PHQ-9 than WSAS and GAD-7, even when these were specified with a cognitive and a somatic factor each [23]. However, such relationships have rarely been investigated using Structural Equation Modeling (SEM).

In the current study, the factor structures of PHQ-9 and GAD-7 will be examined using CFA, where both one-, two- and bifactor models will be tested. Measurement properties across gender, diagnosis, and comorbidity will be evaluated with respect to MI, and the concurrent validity with WSAS will be investigated using SEM. Based on previous research, we hypothesize that two-factor models composed of a cognitive and a somatic factor will fit both instruments best. We expect to achieve scalar invariance across different patient groups for both instruments and that symptoms of depression will predict functional impairment to a greater extent than symptoms of anxiety.

## Method

### Sample

This study was based on data from a psychiatric outpatient clinic in Trondheim, Norway. Patients was referred by general practitioners, or other mental health clinics. Patients completed all instruments before starting treatment. Data was collected using a digital platform from February to November 2020 and informed consent was given electronically. There were no exclusion criteria, but patients diagnosed with some specific disorders (e.g. psychosis and obsessive-compulsive disorder) received outpatient treatment elsewhere and was not represented in this sample. A total of 857 patients consented to participate, 145 declined. Fifteen patients completed the forms twice and the most recent was removed.

Forty-three of the patients did not answer all items. Out of these, 26 did not answer at least one question on one of the three instruments (mean age 33.44 years, 18 women), and were removed. The final sample consisted of 831 patients, with a mean age of 30.03 years (*SD* = 9.99, median = 27, range = 18–72), and 510 were women (61%).

Data for ICD-10 diagnoses was extracted in November 2020. This led to no available diagnosis for some patients that just started therapy. In this sample, 638 (77%) of the patients were diagnosed with an ICD-10 Mental and behavioral diagnosis at the time of data extraction. More women than men had been diagnosed (see Table 1). The most frequent diagnoses were mood disorders (37%) and anxiety disorders (34%). A total of 193 (23%) had comorbid diagnoses (with two or more ICD-10, chapter 5 subsections diagnosis), and of these, 99 (12%) were diagnosed with both a mood disorder (F30-F39) and an anxiety or stress disorder (F40-F49).

**Table 1:** Characteristics of 831 patients on diagnostic, symptoms, and functioning including comparisons between women and men

	Total (***n*** = 831)	Women (***n*** = 510)	Men (***n*** = 321)	t/χ^**2**^	***p***
Demographics					
Age	30.03 (9.99)	29.53 (9.78)	30.81 (10.28)	−1.79	.072
Single	430 (52%)	240 (47%)	190 (59%)	11.61	<.001^***^
Sick leave	211 (25%)	130 (25%)	81 (25%)	0.01	.934
ICD-10 diagnoses					
Undiagnosed	193 (23%)	97 (19%)	96 (30%)	13.10	<.001^***^
Mood disorders, F30-F39	310 (37%)	188 (37%)	122 (38%)	0.11	.740
Anxiety/stress disorders, F40-F48	284 (34%)	194 (38%)	90 (28%)	8.76	.003^**^
Hyperkinetic disorders, F90-F98	134 (16%)	75 (15%)	59 (18%)	1.97	.161
Personality disorders, F60-F69	84 (10%)	61 (12%)	23 (7%)	4.99	.026^**^
Two sections or more	193 (23%)	119 (23%)	74 (23%)	0.01	.926
Sum-score					
PHQ-9	15.82 (5.71)	16.12 (5.61)	15.35 (5.85)	1.89	.059
≥ 10	700 (84.24%)	442 (86.67%)	258 (80.37%)	5.87	.015^*^
GAD-7	12.14 (4.89)	12.66 (4.85)	11.30 (4.83)	3.97	<.001^***^
≥ 10	566 (68.11%)	366 (71.76%)	200 (62.31%)	8.12	.004^**^

*Note.* Results presented include four of the most common ICD-10, chapter 5 sections from the sample. Age, and sum-score are presented as mean (SD). Single, sick leave, ICD-10 diagnoses and over cut-off are presented with number (%)

^*^ *p* < .05, ^**^p < .01, ^***^p < .001

A majority of the patients scored over cut-off for depression and anxiety (≥ 10 for sum-score of PHQ-9 and GAD-7; see Table 1). Women scored statistically significantly higher on GAD-7 and were more associated with scoring greater than cut-off for both PHQ-9 and GAD-7.

Patients with a mood disorder and not an anxiety disorder (*n* = 211) scored significantly higher and more often over cut-off on PHQ-9, and higher on WSAS, than patients with an anxiety disorder and not a mood disorder (*n* = 185; PHQ-9 *t* = 3.35, *p* < .001, *χ*^*2*^ = 6.27, *p* = .012; WSAS *t* = 4.05, *p* < .001). Patients with an anxiety disorder and not mood disorder scored higher on GAD-7, although not significantly more often over cut-off (GAD-7 *t* = − 2.26, *p* = .024, *χ*^*2*^ = 1.72, *p* = .189).

Patients with comorbid diagnosis (*n* = 193) scored significantly higher, and more often over cut-off on all instruments compared with patients diagnosed with only one diagnosis (*n* = 445; PHQ-9 *t* = − 4.95, *p* < .001, *χ*^*2*^ = 15.88, *p* < .001; GAD-7 *t* = − 4.02, *p* < .001, *χ*^*2*^ = 13.61, *p* < .001; WSAS *t* = − 2.60, *p* = .001).

### Instruments

The nine item Patient Health Questionnaire-9 (PHQ-9) measures severity of depression and can also be used as a diagnostic tool [2]. It comes with a diagnostic algorithm but using sum-score and applying a cut-off ≥10 has been suggested to be more sensitive for detecting depression [24]. PHQ-9 uses a 4-point Likert scale ranging from 0 (*not at all*) to 3 (*almost every day*). Its psychometric properties have been widely tested [25–27], and it has demonstrated good properties as a severity measure in a large psychiatric sample [10]. Psychometric properties of the Norwegian version have been tested with adolescents and adult women with and without eating disorders [28, 29].

The seven item Generalized Anxiety Disorder scale-7 (GAD-7) [3] was developed to detect and measure severity of generalized anxiety disorder. However, it has been demonstrated to perform well as a measure of other anxiety symptoms as well [16, 30]. The GAD-7 uses an identical 4-point Likert scale as the PHQ-9. It is considered to be a reliable and valid measure of anxiety symptoms in heterogenous psychiatric outpatients, amongst others in Norway and the U.S. [14, 16]. Both PHQ-9 and GAD-7 are available in several languages [31].

The Work and Social Adjustment Scale (WSAS) [21] measures functional impairment. It consists of five items that assess impairment of daily functioning (work, home chores, social leisure, private leisure, and relationships) that are rated on a 9-point Likert scale from 0 (*not at all impaired*) to 8 (*very severely impaired*). The psychometric properties of WSAS have been demonstrated in various studies, in a Norwegian outpatient setting [22] and in England, where it is suggested to be a good complement to PHQ-9 and GAD-7 [32].

### Statistical analysis

Stata [33] was used for data preparation and testing group differences. Mplus version 8.4 [34] was used for CFA, MI and SEM. Missing items were less than 0.01% on all variables. Little’s MCAR test showed non-significant results (PHQ-9 *p* = .88, GAD-7 *p = .*78*,* WSAS *p = .*73), indicating that data were missing completely at random. No imputations were done.

Weighted Least Squares Means and Variance adjusted (WLSMV) estimator was used [35], as it is less prone to bias than other estimators for ordinal data [36]. Several fit indices were used [17]: *χ*^2^ as a measure of absolute fit, Root Mean Square Error of Approximation (RMSEA) for parsimony correction, and the comparative fit indices Comparative fit index (CFI) and Tucker-Lewis index (TLI) [37]. Thresholds close to or below .06 for RMSEA and above .95 CFI and TLI were used to indicate good fit [38].

A bifactor model was specified using the bifactor-(S – 1) modification, specified with a specific factor, and a reference domain [13]. Bifactor-(Sc – 1) was estimated with a specific cognitive group factor and by using the somatic domain as reference. Bifactor-(Ss – 1) was estimated with a specific somatic group factor and by using the cognitive domain as reference.

Internal consistency was measured with composite reliability, which has been proposed as a superior alternative to other measures [39]. A value between .7 and .9 was used for satisfactory internal consistensy. Discriminant validity was calculated with confidence intervals in CFA, using standardized Upper Limit 95% confidence intervals (UL) for correlation between the factors. UL < 0.8 indicates no problem, 0.8–0.9 indicates marginal problems, 0.9–1.0 indicates moderate problem and above 1.0 indicates severe problems [39].

Omega hierarcical was estimated [40], and omega hierarchical above .8 was interpreted to indicate a primarily one-dimensional construct [41]. Additionally, one-dimensionality was also interpreted if omega hierarchical for the general factor was over .7, percent of uncontaminated correlations (PUC) was lower than .8 and explained common variance (ECV) of the general factor was over .6 [41].

Measurement Invariance (MI) was evaluated sequentially, for configural, metric and scalar invariance, where each step implied more equality constraints. Configural invariance was achieved if the pattern of free and fixed loadings across gender was equivalent, i.e. number of factors and indicator-factor patterns were considered the same across men and women [17]. If configural invariance was supported, metric invariance was tested next, where factor loadings were constrained equally. If metric invariance was achieved, scalar invariance was evaluated by constraining item thresholds to be equal across the groups. Scalar invariance implies that differences in latent means are not biased and may be considered to be true differences between genders. We followed the recommendations by Millsap and Yun-Tein [42] and Pendergast with colleagues [43] for testing MI with ordered-categorical measures. The Mplus DIFFTEST function was used for comparison of model fit [33]. However, using ΔCFI ≥ − .01 and ΔRMSEA <.015 has been suggested to be superior for evaluate MI, than relying on non-significant ∆𝜒^2^ [44]. Thus, ΔCFI and ΔRMSEA was used for threshold guidance. For concurrent validity, latent path modeling with SEM was used with bifactor-(S – 1).

## Results

### Factor structure

Unitary factor solution of the PHQ-9 resulted in non-satisfactory fit statistics (model 1 in Table 2). PHQ-9 demonstrated better fit statistics with a two-factor solution and was accepted without modifications (model 2 in Table 2). The two-factor solution of PHQ-9 consisted of a cognitive factor of depression: PHQc (items 1, 2, 6, & 9), and a somatic factor of depression: PHQs (items 3, 4, 5, 7, & 8). Both PHQ-9 bifactor-(S – 1) models resulted in similar goodness of fit as the two-factor solution (model 3 and 4 in Table 2).

**Table 2:** Goodness of fit for Confirmatory factor analysis of PHQ-9, GAD-7 and WSAS (*n* = 831)

Model	𝜒^**2**^	df	RMSEA [90% CI]	CFI	TLI
*Total*					
1. PHQ-9 single factor	341.080^***^	27	.118 [.107–.130]	.937	.916
2. PHQ-9 two-factor	105.070^***^	26	.060 [.049–.073]	.984	.978
3. PHQ-9 bifactor-(Sc – 1)	101.667^***^	23	.064 [.052–.077]	.984	.975
4. PHQ-9 bifactor-(Ss – 1)	103.436^***^	22	.067 [.054–.080]	.984	.973
5. GAD-7 single factor	183.117^***^	14	.121 [.105–.136]	.976	.964
6. GAD-7 single factor mod.^1^	50.288^***^	11	.066 [.048–.084]	.994	.989
7. GAD-7 two-factor	61.920^***^	13	.067 [.051–.085]	.993	.989
8. GAD-7 two-factor mod.^2^	45.815^***^	12	.058 [.041–.077]	.995	.991
9. GAD-7 bifactor-(Sc – 1)	42.805^***^	10	.063 [.044–.083]	.995	.990
10. GAD-7 bifactor-(Ss – 1)	50.288^***^	11	.066 [.048–.084]	.994	.989
11. WSAS single factor	138.321^***^	5	.179 [.154–.205]	.953	.906
12. WSAS mod.^3^	14.235^***^	4	.055 [.026–.088]	.996	.991
13. WSAS mod.^3^ & PHQ-9 bifactor-(Sc – 1)	274.640^***^	70	.059 [.052–.067]	.976	.968
14. WSAS mod.^3^ & PHQ-9 bifactor-(Ss – 1)	274.386^***^	69	.060 [.053–.067]	.975	.968
15. WSAS mod.^3^ & GAD-7 bifactor-(Sc – 1)	180.710^***^	47	.059 [.050–.068]	.985	.979
16. WSAS mod.^3^ & GAD-7 bifactor-(Ss – 1)	195.707^***^	48	.061 [.052–.070]	.983	.977

*Note.* df = degrees of freedom. Bifactor-(Sc – 1): cognitive group factor, with somatic domain as reference. Bifactor-(Ss – 1): somatic group factor, with cognitive domain as reference. ^1^Items 4, 5, and 6 correlated residuals. ^2^Items 2 and 3 correlated residuals. ^3^Items 3 and 5 correlated residuals. ^***^p < .001

A unitary factor solution for GAD-7 showed poor model fit (model 5 in Table 2). GAD-7 was also tested for a unitary factor solution, with a proposed somatic factor (items 4, 5, & 6) as correlated residuals (model 6 in Table 2). This latter solution provided acceptable model fit, although over the RMSEA treshold of ≤ .06. A two-factor solution yielded similar model fit as model 6 (model 7 in Table 2). Modification indices indicated a substantial residual covariance between item 2 and item 3 (Standardized Expected Parameter Change index [Stdyx E.P.C] .492) of the two-factor solution. Allowing these residuals to covary (*δ* = .34, *p* < .001) resulted in an overall good fit, and this model was accepted (model 8 in Table 2). The model consisted of a cognitive factor of anxiety: GADc (items 1, 2, 3, & 7; with correlated residuals between item 2 & 3) and a somatic factor of anxiety: GADs (items 4, 5, & 6). Both GAD-7 bifactor-(S – 1) resulted in similar goodness of fit as the two-factor solution (model 9 and 10 in Table 2).

WSAS was also tested with CFA, to assess its suitability to evaluate concurrent validity of PHQ-9 and GAD-7. A unitary factor model resulted in unsatisfactory fit statistics (model 11 in Table 2). Modification indices indicated a substantial residual covariance between item 3 & item 5; Stdyx E.P.C .51). Allowing error terms to correlate (Stdyx total *δ* = .37, *p* < .001) yielded a good fit (model 12 in Table 2). CFA with WSAS correlated with the PHQ-9 and GAD-7 bifactor-(S – 1) demonstrated good fit statistics for the total sample (model 13–16 in Table 2).

Standardized factor loadings for PHQc were between *λ* = .91 (item 2) and *λ* = .70 (item 9), and for PHQs between *λ* = .77 (item 4) and *λ* = .60 (item 8). For GADc it varied between *λ* = .88 (item 1) and *λ* = .73 (item 7), and for GADs it varied between *λ* = .85 (item 4) and *λ* = .54 (item 6). Composite reliability for PHQc was .87 and .80 for PHQs. For GADc it was .90 and for GADs .73. All factor loadings were above .5 and composite reliability were greater than .7, thus demonstrating acceptable loadings and internal consistensy reliability between indicator variables. The correlation between the factors in PHQ-9 and GAD-7 were all strong (PHQc with PHQs: *φ* = .74, S.E. = .03, UL = .79; GADc with GADs: *φ* = .80, S.E. = .03, UL = .85). The cognitive factors demonstrated weaker correlation with each other (*φ* = .67, S.E. = .03, UL = .72) than the somatic factors with each other (*φ* = .84, S.E. = .03, UL = .90). The weakest correlations were between the PHQc with GADs (*φ* = .57, S.E. = .04, UL = .64), and PHQs with GADc (*φ* = .67, S.E. = .03, UL = .73).

Test for dimensionality resulted in mainly one-dimensional results for the general factors, with some minor issues (see Table 3). Omega hierarchical for PHQ-9 bifactor-(Sc – 1) were below .8, but the PUC and ECV-values justified a one-dimensional interpretation, albeit with some indication of multidimensionality (omega hierarchical = .78, PUC = .83, ECV = .76). Comparable results were found for PHQ-9 bifactor-(Ss – 1) (omega hierarchical = .77, PUC = .72, ECV = .78), and for GAD-7 bifactor-(Sc – 1) (omega hierarchical = .76, PUC = .71, ECV = .75). For GAD-7 bifactor-(Ss – 1) the omega hierarchical was above .8, and thus interpreted as mainly one-dimensional (omega hierarchical = .85, PUC = .86, ECV = .85). The mean omega hierarchical was .78 for PHQ-9, and .81 for GAD-7.

**Table 3:** Standardized factor loadings and omega hierarchical for PHQ-9 and GAD-7

Items		GeneralC	SpecificC	GeneralS	SpecificS	General mean
phq1	Little interest or pleasure […]	.600	.509	.798		.699
phq2	Feeling down, depressed, or hopeless	.599	.657	.425		.512
phq3	Trouble falling […] asleep, or sleeping too much	.775		.591	.500	.683
phq4	Feeling tired or having little energy	.661		.913	.457	.787
phq5	Poor appetite or overeating	.631		.441	.375	.536
phq6	Feeling bad about yourself […]	.667	.450	.519		.593
phq7	Trouble concentrating on things […]	.587		.755	.464	.671
phq8	Moving or speaking slowly […] or the opposite[…]	.631		.454	.460	.543
phq9	Thoughts that you would be better off dead […]	.492	.507	.695		.594
	*PHQ-9 Omega Hierarchical*	.784	.392	.770	.302	.777
gad1	Feeling nervous […]	.712	.452	.851		.782
gad2	Not able to stop worrying	.662	.605	.887		.775
gad3	Worrying too much about different things	.665	.613	.894		.780
gad4	Having trouble relaxing	.855		.674	.360	.765
gad5	Being so restless that it is hard to sit still	.643		.484	.683	.564
gad6	Becoming easily annoyed or irritable	.542		.442	.249	.492
gad7	Feeling afraid […]	.604	.371	.716		.660
	*GAD-7 Omega Hierarchical*	.761	.338	.850	.294	.806

*Note. GeneralC* General factor using somatic domain as reference, *SpecificC* Specific cognitive factor, *GeneralS* General factor using cognitive domain as reference, *SpecificS* Specific somatic factor

### Measurement invariance

Scalar invariance was achieved across genders, diagnoses, and comorbidity for all bifactor-(Sc – 1) solutions of PHQ-9 and GAD-7 (Table 4). Thus, with cut-off values of ΔCFI ≥ − .01 and ΔRMSEA <.015, this demonstrated equality of factor loadings, equality of indicator tresholds, and equality of indicator residuals. PHQ-9 for patients with a diagnosis of depression versus patients with an anxiety disorder diagnosis demonstrated issues with achieving configural invariance according to the RMSEA value. However, the CFI-value was above the treshold and interpreted as supporting configural invariance. Latent mean differences (LMD) using bifactor-(Sc – 1) resulted in significantly higher scores on PHQ-9 for women (LMD = .38, SE = .09, *p* < .001), and patients with comorbidity (LMD = .40, SE = .11, *p* < .001), but no significant differences between depression and anxiety diagnoses were found (LMD = .21, SE = .12, *p* = .083). Comparable results were found for GAD-7, with significantly higher scores for women (LMD = .37, SE = .09, *p* < .001), patients with comorbidity (LMD = .37, SE = .11, *p* < .001), with non-significant results for depression vs. anxiety (LMD = −.22, SE = .17, *p* = .115).

**Table 4:** Measurement invariance using bifactor-(Sc – 1) solution of PHQ-9 and GAD-7

	χ^**2**^ (df)	CFI	RMSEA [90% CI]	∆ χ^**2**^ (df)	***p***	∆CFI	∆RMSEA
*Gender*							
*PHQ-9*							
Configural	120.690 (46)	.985	.063 [.049–.076]	–	–	–	–
Metric	117.193 (57)	.988	.050 [.037–.063]	8.210 (11)	.694	.003	−.013
Scalar	130.459 (73)	.989	.044 [.031–.055]	18.611 (16)	.289	.001	−.006
*GAD-7*							
Configural	59.039 (20)	.994	.069 [.049–.089]	–	–	–	–
Metric	70.058 (29)	.994	.058 [.041–.076]	17.209 (9)	.046	.000	−.011
Scalar	68.998 (41)	.996	.041 [.023–.057]	4.492 (12)	.973	.002	−.017
*Depression* vs. *Anxiety*							
*PHQ-9*							
Configural	108.401 (46)	.969	.083 [.063–.103]	–	–	–	–
Metric	128.598 (57)	.980	.063 [.048–.077]	26.805 (11)	.005	.011	−.020
Scalar	144.209 (73)	.980	.055 [.042–.069]	20.402 (16)	.203	.000	−.008
*GAD-7*							
Configural	26.786 (20)	.998	.041 [.000–.079]	–	–	–	–
Metric	38.262 (29)	.997	.040 [.000–.072]	12.163 (9)	.204	−.001	−.001
Scalar	58.513 (41)	.994	.047 [.012–.072]	20.473 (12)	.059	−.003	.007
*Comorbid* vs. *single diagnsosis*							
*PHQ-9*							
Configural	105.079 (46)	.984	.063 [.047–.080]	–	–	–	–
Metric	128.598 (57)	.980	.063 [.048–.077]	26.805 (11)	.005	−.004	.000
Scalar	144.209 (73)	.980	.055 [.042–.069]	20.402 (16)	.203	.000	−.008
*GAD-7*							
Configural	44.238 (20)	.996	.062 [.037–.086]	–	–	–	–
Metric	47.574 (29)	.997	.045 [.019–.067]	8.708 (9)	.465	.001	−.017
Scalar	59.560 (41)	.997	.038 [.012–.057]	13.513 (12)	.333	.000	−.007

*Note.* ΔCFI ≥ − .01 and ΔRMSEA < .015 indicates established MI. Gender (*n* = 831), depression/anxiety (*n* = 396), comorbidity/no comorbidity (*n* = 638)

### Concurrent validity with WSAS

**Fig. 1:**
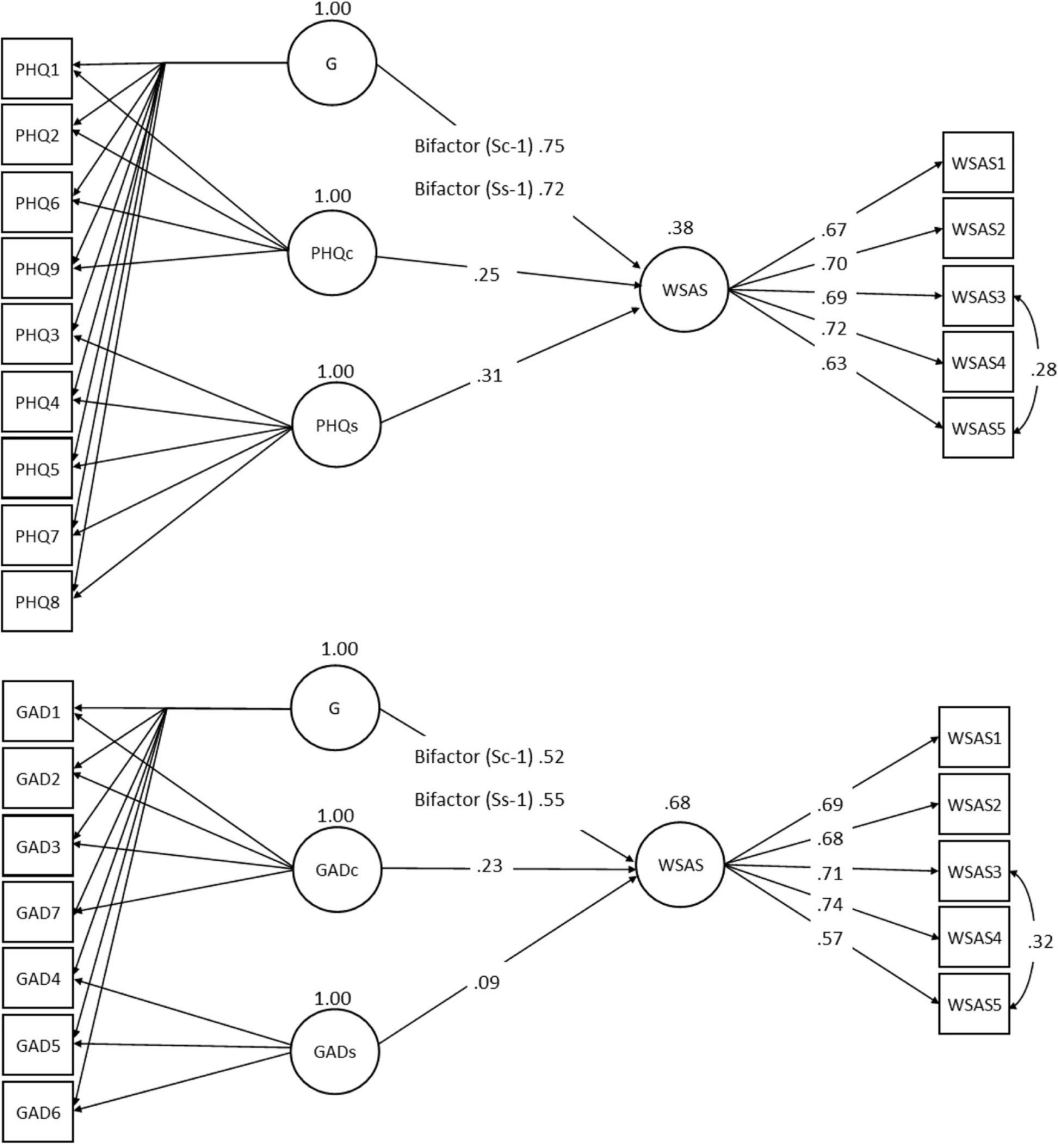
Standardized results from Latent path models (*n* = 831), where all loadings and paths are significant at *p* < .001, except WSAS regressed on GADs (*p* = .038)

WSAS regressed on bifactor-(S – 1) models of PHQ-9 and GAD-7 each resulted in significant coefficients for the full sample (see Fig. 1). The general factors demonstrated stronger associations with functional impairment than the cognitive and somatic factors, and PHQ-9 demonstrated a stronger association with functional impairment than GAD-7 (WSAS regressed on general factor mean PHQ-9 *γ* = .74, *r*^2^ = .62; WSAS regressed on general factor mean GAD-7 *γ* = .54, *r*^2^ = .32). WSAS regressed on the general bifactor-(Sc – 1), resulted in higher associations with PHQ-9 (women *γ* = .82, *r*^2^ = .78, men *γ* = .70, *r*^2^ = .53; anxiety *γ* = .52, *r*^2^ = .65, depression *γ* = .41, *r*^2^ = .49; no comorbidity *γ* = .74, *r*^2^ = .61, comorbidity *γ* = .62, *r*^2^ = .53) than GAD-7 (women *γ* = .54, *r*^2^ = .39, men *γ* = .50, *r*^2^ = .28; anxiety *γ* = .67, *r*^2^ = .46, depression *γ* = .44, *r*^2^ = .24; no comorbidity *γ* = .52, *r*^2^ = .31, comorbidity *γ* = .39, *r*^2^ = .21).

## Discussion

The aim of this study was to test the factor structure and measurement invariance of PHQ-9 and GAD-7 in a heterogeneous psychiatric outpatient sample. We also examined the concurrent validity of PHQ-9 and GAD-7 with functional impairment, measured with WSAS, across gender. Firstly, the results supported a two-factor solution for both PHQ-9 and GAD-7, consisting of a cognitive and a somatic factor for each measure. This finding corresponds with previous research with heterogenous outpatient samples [11, 16]. However, tests for dimensionality of the instruments indicated a general factor, which demonstrated acceptable fit statistics, in accordance with previous studies [12].

Secondly, the bifactor solutions PHQ-9 and GAD-7 achieved scalar invariance across gender, diagnosis, and comorbidity which supports that both instruments measure the same construct for different patient groups, and hence are suitable for comparing differences across these.

Thirdly, all factors were significantly associated with functional impairment, with the general factors accounting for most of the variance compared to the cognitive and somatic factors. However, symptoms of depression demonstrated stronger associations with functional impairment than symptoms of anxiety. Thus, PHQ-9 and GAD-7 demonstrate support for a general factor, albeit with cognitive and somatic subcomponents, when used in heterogenous psychiatric outpatients.

The background of this study was limited research regarding properties of the PHQ-9 and GAD-7 in heterogenous clinical populations. Non-clinical populations may display greater variance in item scores and therefore load on a single factor [8]. In contrast, patients in the present study were assessed prior to psychiatric treatment, and therefore the sample represents a more heterogeneous population. Previous research has advised against multidimensional solutions of these instruments, due to strong factor correlations [11]. Other studies have justified using a sum-score for PHQ-9 and GAD-7 using the extracted factors from an EFA in a bi-factor model [9]. However, such model may create a risk of overfitting the data, and the results could be seriously affected by captured noise [45].

A strength in present study was examining the factor structure a-priori, using the same factor structure specified using a similar population [7]. Additionally, we specified these underlying subdimensions using a modified bifactor, well suitable to our data [13]. However, in the present study patients completed assessment before treatment, and we therefore examined a more heterogenous population. Thus, the present study adds to the knowledge of how to properly specify a bifactor model in studies with heterogenous patients initiating treatment.

Some modifications were made to the two-factor solutions, based on both statistical properties and theoretical justifications. We decided to let the residuals (item 2 and 3 covering *Not being able to stop/control worrying, and Worrying too much*) in GAD-7 covary due to their similarities, and let residual covary (item 3 and 5, covering *Impaired social activities, and Impaired close relationships*) in WSAS, which corroborates with previous results from Norwegian outpatients [22]. The suggested unitary factor solution with correlated residuals regarding GAD-7 [14, 15] could be criticized for overlooking theoretical reasoning. We argue that the correlations between these (items 4, 5, and 6 covering *Trouble relaxing*, *Being restless* and *Being* e*asily annoyed*) are essential parts of the latent anxiety construct (i.e. a somatic factor), hence, not to be viewed as misfits in the two-factor model. But the moderate problem with discriminate validity between this somatic factor of anxiety and the somatic factor of depression indicate that these constructs are not very well separatable. And the low factor loadings, and a potential crossloading (i.e. GAD-7 item 5 and PHQ-9 item 8 both deal with restlessness), mean that these factors must be handled cautiously. The high correlations can potentially lead to multicollinearity problems if used simultanously, e.g. in multiple regression. If these instruments would be further revised, our recommendation would be to investigate GAD-7 item 4, 5, 6, i.e. the somatic factor of anxiety. Regarding the cognitive factors, the weaker correlations between PHQc and GADc implies that these two factors explains two different constructs, i.e. a cognitive aspect of depression and anxiety each.

To the best of our knowledge, no previous studies have to the same extent examined the association of the factor structure of PHQ-9 and GAD-7 on functional impairment across patient groups in a heterogenous psychiatric outpatient population. The results indicate justification of using these instruments as one-dimensional in clinical settings for measuring symptom severity. However, the results suggest the importance of specifying the underlying factor structure when precise estimates are needed. Further, factorization of these instruments will assess symptom severity measured by a latent general factor. These factors are more robust for comparisons across groups, but the instruments may also be valuable as diagnostic tools, or for single item assessment. For example, we found that PHQ-9 item 9 which assesses suicidal thoughts loaded the general factor below .6, which still has a high clinical value.

Several limitations to this study should be noted. The results are limited by the observational nature of the study. Although few patients declined participation, we were not able to control their reasons nor background data due to research ethical concerns for patients who did not consent to participation. Furthermore, patients were diagnosed in a non-controlled environment, hence, no inter-rater reliability was available, and follow-up assessment is not reported.

Another noteworthy point is that when estimating the bifactor-(S – 1), the general factor was defined by the reference domain. MI and LMD was estimated using somatic domain as reference, thus the scores of the general factor could be interpreted as somatic symptoms corrected for measurement error. Thus, MI and LMD could also be calculated with the cognitive domain as a reference. It is suggested for further studies, to do multiple sampling for overcoming the problems with anomalous results using symmetric bifactors if such solution are preferred. However, a symmetrical bifactor will also create ambiguous interpretations [13, 45].

Additionally, using a longitudinal design could determine the suitability of using the instruments over time. Examining for example individual differences and clinical subgroups over time would improve the clinical utility of these instruments in treatment of mental illness.

## Conclusion

The results of this study show that PHQ-9 and GAD-7 may be conceptualized as one-dimensional instruments, with underlying subdimensions of both cognitive and somatic factors. We found support for measurement invariance across gender, diagnostic subgroups and comorbidity, which means that the instruments are interpreted equally among these groups of patients. The higher associations between functional impairment and symptoms of depression highlights the importance with this relation.

Thus, one-dimensionality was supported, and an aggregated score can be justified in clinical settings. However, when precise estimation is needed, such as in psychometric studies with heterogeneous psychiatric populations, our results suggest that the underlying subdimensions should be specified. In conclusion, our study lends further support for the use of PHQ-9 and GAD-7 for assessment of symptoms of depression and anxiety in patients with mental illness.

## Data Availability

Data are available from the corresponding author on reasonable request.

## Abbreviations

PHQ-9: The nine item Patient Health Questionnaire-9
GAD-7: The seven item Generalized Anxiety Disorder scale-7
EFA: Exploratory Factor Analysis
CFA: Confirmatory Factor Analysis
MI: Measurement Invariance
WSAS: The Working Social Adjustment Scale
SEM: Structural Equation Modeling
WLSMV: Weighted Least Squares Means and Variance adjusted
RMSEA: Root Mean Square Error of Approximation
CFI: Comparative Fit Index
TLI: Tucker-Lewis Index
UL: Upper Limit 95% confidence interval
Stdyx E.P.C: Standardized Expected Parameter Change index
PUC: Percent of Uncontaminated Correlations
ECV: Explained Common Variance
LMD: Latent mean differences
